# Maximum Entropy Estimates of Hubble Constant from Planck Measurements

**DOI:** 10.3390/e27070760

**Published:** 2025-07-16

**Authors:** David P. Knobles, Mark F. Westling

**Affiliations:** 1 The Platt Institute for Cosmology and Nuclear Physics, Austin, TX 78731, USA; 2 Meta, Menlo Park, CA 94025, USA; markwestling@meta.com

**Keywords:** maximum entropy, cosmology, Planck, dark matter, Hubble constant, Hubble tension

## Abstract

A maximum entropy (ME) methodology was used to infer the Hubble constant from the temperature anisotropies in cosmic microwave background (CMB) measurements, as measured by the Planck satellite. A simple cosmological model provided physical insight and afforded robust statistical sampling of a parameter space. The parameter space included the spectral tilt and amplitude of adiabatic density fluctuations of the early universe and the present-day ratios of dark energy, matter, and baryonic matter density. A *statistical temperature* was estimated by applying the equipartition theorem, which uniquely specifies a posterior probability distribution. The ME analysis inferred the mean value of the Hubble constant to be about 67 km/sec/Mpc with a conservative standard deviation of approximately 4.4 km/sec/Mpc. Unlike standard Bayesian analyses that incorporate specific noise models, the ME approach treats the model error generically, thereby producing broader, but less assumption-dependent, uncertainty bounds. The inferred ME value lies within 1σ of both early-universe estimates (Planck, Dark Energy Signal Instrument (DESI)) and late-universe measurements (e.g., the Chicago Carnegie Hubble Program (CCHP)) using redshift data collected from the James Webb Space Telescope (JWST). Thus, the ME analysis does not appear to support the existence of the Hubble tension.

## 1. Introduction

Bayesian methods have played an important role in solving statistical inverse problems in the physical sciences. Research areas where *orthodox* Bayesian methods have been useful include geophysics [1,2,3] and condensed matter [4,5]. In the Bayesian approach, it is necessary to construct a likelihood where prior assumptions are required in, for example, the statistics of an error function that incorporate model and data errors. Therefore, from Bayes’ rule, the resulting posterior probability distribution (PPD) has the imprint of any biased prior assumptions made in the construction of the likelihood. In contrast, the maximum entropy (ME) concept introduced by Jaynes [6,7] offers an alternative to a strictly Bayesian-derived PPD in that the resulting ME PPD provides the least-informative or conservative model parameter probability densities. *Jaynes argued that the ME is best used for constructing prior probability distributions with minimal assumptions, thereby making it a valuable tool in Bayesian analyses* [8]. While setting prior distributions can be useful for certain applications, we agree with the sentiment expressed in [9] that the ME is significantly more general and useful when formulated with a *relative* entropy function [10], which results in full consistency with Bayes’ rule. In addition, an ME likelihood is derived using the *equipartition theorem*, which is consistent with a PPD that provides the most conservative degree of information on the state of knowledge of a system described by a physical model, given testable data.

**Figure 1:**
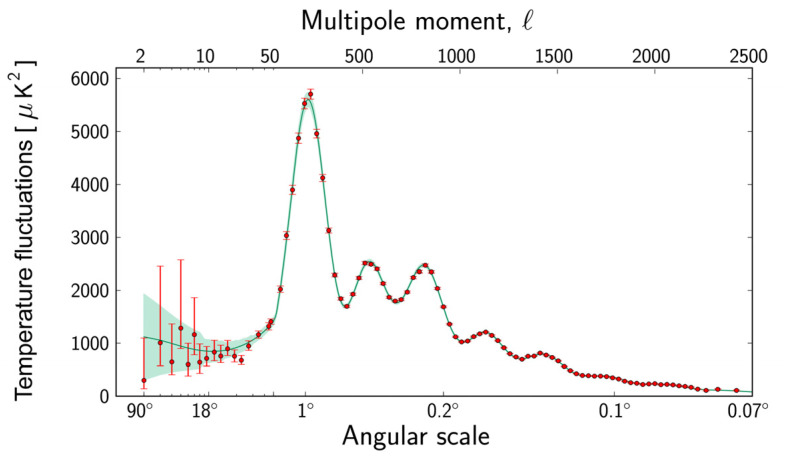
Power spectrum for temperature fluctuations. Graph made available online: https://www.esa.int/ESA_Multimedia/Images/2013/03/Planck_Power_Spectrum (accessed on 6 January 2025), courtesy of the ESA and the Planck Collaboration.

As an application, the ME methodology can be utilized to analyze the multipole power spectra of temperature anisotropies measured by the European Space Agency Planck satellite [11,12] for the purpose of conservatively inferring information on model cosmological parameter values, such as the present-day percentages of matter and dark energy and the adiabatic density fluctuations in the primordial universe. The measurements by the Planck satellite significantly extend the multipole scale and resolution of the power spectra relative to its critically important predecessor, the Wilkinson Microwave Anisotropy Probe (WMAP) [13]. Figure 1 shows the temperature anisotropies in the cosmic microwave background power spectrum measured by the Planck satellite. The top horizontal axis is a multipole scale that is inversely proportional to the angular scale on the bottom horizontal axis. The red dots are measurements made with Planck and the green curve represents the best fit of the ΛCDM model [14,15,16,17], also referred to as the *standard model of cosmology.* In the ΛCDM model, *lambda* and *CDM* refer to dark energy [18,19], or the cosmological constant, and cold dark matter [20,21], respectively. A damped acoustic wave, driven by quantum adiabatic density fluctuations that start during inflation, occurs in a viscous fluid that includes photons and baryonic plasma [22]. The acoustic signatures were *frozen* on the last scattering surface and are evident in the peak and null structure of the multipole power spectra. The region of l<30 or from 6 degrees to 90 degrees, known as the Sachs–Wolfe region [23], is where there are uncertainties in both the data and the theory.

The ME method provides the proper treatment of the model error and its effects on parameter ambiguities and uncertainty. The cosmological model used in the current analysis is an approximate approach referred to as the hydrodynamical model (HM), previously discussed by Hu and Sugiyama [24] and Weinberg [25]. In the current analyses, we only considered the region of l>30. The remainder of this paper is organized as follows. Section 2 presents the ME method and Section 3 presents the HM and parameter space and the assumed parameter constraints used to simulate the power spectra of the CMB temperature anisotropies. Section 4 presents the results and Section 5 and Section 6 provide a brief discussion and the conclusions, respectively.

## 2. Maximum Entropy

Jaynes [6,7] argued that the Shannon entropy [26,27] developed in information theory and the entropy that appears in the field of statistical mechanics possess the same mathematical logic. Namely, entropy is not just a term that appears in the second law of thermodynamics for a closed system in a thermal equilibrium, but rather is a more general mathematical concept. In this paper, this logic was utilized to infer probability distributions for model parameters Θ that appear in a cosmological model for the expansion of the universe from measured temperature anisotropy measurements of the cosmic microwave background radiation.

The development of the ME formalism began by introducing the Kullback *relative* entropy [10] functional S:

(1) $$S=-\int d\Theta \;𝒫\left(\Theta |D\right)\ln\frac{𝒫(\Theta |D)}{𝒫\left(\Theta \right)},$$

where $𝒫\left(\Theta |D\right)$ is a conditional posterior probability distribution (PPD), $𝒫\left(\Theta \right)$ is the model prior distribution, and D is the measured data. Note that (1) is the same as for statistical mechanics except that the Boltzmann constant $k_{B}$ is set to unity. The reason one wants to introduce $𝒫\left(\Theta \right)$ is to restrict statistical sampling to physical portions of the parameter space. The objective is to find a conditional probability density $𝒫\left(\Theta |D\right)$ subject to the following constraints:

(2) $$\int d\Theta \;𝒫\left(\Theta |D\right)=1$$

and the average error satisfying

(3) $$<E>=\int d\Theta \;E\;\left(\Theta ,D\right)𝒫\left(\Theta |D\right).\;$$

Lagrange multipliers, α and β, are introduced to find $𝒫\left(\Theta |D\right)$, which extremizes S subject to the conditions in (2) and (3). The condition of extremization is

(4) $$\delta \left\{\int d\Theta \;[\alpha \;𝒫\left(\Theta |D\right)+\beta E\left(\Theta ,D\right)\;𝒫(\Theta |D)-𝒫\left(\Theta |D\right)\;\ln\frac{𝒫(\Theta |D)}{𝒫\left(\Theta \right)}]\right\}=0$$

or

(5) $$\int d\Theta \;\left[\left\{\alpha -1+\beta E\left(\Theta ,D\right)-\ln\frac{𝒫\left(\Theta |D\right)}{𝒫\left(\Theta \right)}\;\right\}\delta 𝒫\left(\Theta |D\right)\right]=0.$$

Since δP is arbitrary, one finds

(6) $$\alpha -1+\beta E\left(\Theta ,D\right)-\ln\frac{𝒫\left(\Theta |D\right)}{𝒫\left(\Theta \right)}=0$$

or, equivalently,

(7) $$\frac{𝒫(\Theta |D)}{𝒫\left(\Theta \right)}=\exp\left(\left(\alpha -1+\beta E\left(\Theta ,D\right)\right)\right).$$

It is customary to define the partition function Z as

(8) $$Z\left(\beta \right)=\exp\left(1-\alpha \right)=\int d\Theta 𝒫\left(\Theta \right)\exp\left(-\beta E\left(\Theta ,D\right)\right).$$

Using (2) and (3), the PPD becomes

(9) $$𝒫\left(\Theta |D\right)=\frac{𝒫\left(\Theta \right)}{Z\left(\beta \right)}\exp\left(-\beta E\left(\Theta ,D\right)\right)$$

where the average error is

(10) $$<E\left(\beta \right)>=\int d\Theta \;E\left(\Theta ,D\right)\frac{𝒫\left(\Theta \right)}{Z\left(\beta \right)}\exp\left(-\beta E\left(\Theta ,D\right)\right).$$

If $Z\left(\beta \right)$ and $\exp\left(-\beta E\left(\Theta ,D\right)\right)$ are identified as $𝒫\left(D\right)$ and the likelihood $𝒫\left(D|\Theta \right)$, respectively, then one can observe that (9) is Bayes’ rule. We will refer to (9) and (10) as the model space representation of the ME.

Jaynes did not explicitly discuss how to determine β in practice. A simple examination of (10) shows that, if one sets $<E>$ to the *global minima* $E\left(\hat{\Theta },D\right)$, $\hat{\Theta }\in \;\Theta \;$ such that $E\left(\hat{\Theta },D\right)\leq \;E\left(\Theta ,D\right)$ for all Θ, then β tends to ∞, which gives marginal distributions that are δ-like functions. On the other hand, if $<E>$ is set to the average error function in the volume of the *N*-dimensional parameter space, β tends to zero, which gives flat marginal distributions. To overcome this problem, the authors in [28] formulated *the data ensemble approach* to the ME, which originated with a methodology to train neural networks on multiple data sets [29,30,31]. The data, as opposed to the model formulation of the ME, afforded an estimate for $<E>$, from which (10) could be solved for β. Instead of using (10) to evaluate $<E>$ for a single data set D, it is assumed that each measurement is a random draw from a data ensemble possessing a continuum of samples $D^{\prime }$, which permits an approximate evaluation of $<E_{j}>$ for the jth data sample:

(11) $$<E_{j}>\approx \;\int dD^{\prime }\;𝒫\left(D^{\prime }\;|{\hat{\Theta }}_{j}\;\right)E\left({\hat{\Theta }}_{j},D^{\prime }\right).$$

If one has a finite number of random measurements $N_{k}$, then

(12) $$<E_{j}>\approx \frac{1}{N_{k}}\;\sum_{k=1}^{N_{k}}E\left({\hat{\Theta }}_{j},\;D_{k}\right)\;$$

which permits an estimate for $\beta_{j}\;$($j=1,2,\cdots ,\;N_{k}$) using (10). This concept was recently applied to multiple merchant ships in a shipping lane on the continental New England shelf [32]. However, for the case where $N_{k}$ is small or there is only a single data sample, as was the case for the current study, a different approach is required. For the CMB, even though what is observed today was a random process, one only observes a single realization in the data, and thus, (12) is not suitable.

Instead of trying to solve (10) for β, we used an analogy with classical statistical mechanics that relates β to a statistical temperature *𝒯*. We remark here that *𝒯* has nothing to do with the temperature *T* of a physical system, including the universe. One first identifies the error function E as an energy, and $\exp\left(-\beta E\right)$ as the Boltzmann factor. Further, one can view the *N*-dimensional space as a system that is in thermodynamic equilibrium with a heat bath at 𝒯. In such a case, we used the Helmholtz free energy equation for the free energy F, the entropy S, 𝒯, and the internal energy U:

(13) F=U−𝒯S

and

(14) $$U=<E>=-\frac{\partial \;\ln Z}{\partial \;\beta }.\;$$

Continuing with Jaynes philosophy, one can identify F and U as the free Helmholtz error and the internal error, respectively. If we identify $<E>=U$, then *𝒯* may be defined as $\beta ={𝒯}^{-1}$, yielding the following:

(15) $$𝒫\left(\Theta |D\right)=\frac{𝒫\left(\Theta \right)}{Z\left(𝒯\right)}\exp\left(-\frac{1}{𝒯}E\left(\Theta ,D\right)\right).$$

Thus, if one identifies the exponential term as a likelihood 𝒫(D|Θ) and knowing that $𝒫\left(D\right)=Z\left(𝒯\right)$, (15) is once again Bayes’ rule.

Equation (15) seems to suggest that one has simply replaced the problem of estimating β with the problem of estimating 𝒯. To address this apparent dilemma, it is instructive to derive the *equipartition theorem* from the first principles, and this is achieved in Appendix A. The result is

(16) $$<E>=\sum <\varepsilon_{i}>=\frac{N}{2}\;𝒯.\;$$

Equation (16) allows us to identify *<E>* as the computed global error minimum, and then use 𝒯, which uniquely specifies 𝒫(Θ|D). The idea of using (16) was first reported by Nuttall et al. [33] in the analysis of merchant ship noise for the information contained on parameter values that characterize a multilayer seabed. The applicability of the equipartition theorem in this context is heuristic, drawing from analogies with classical statistical mechanics. Although the CMB parameter space is not a literal thermodynamic system, the ME framework uses the theorem as a proxy to derive a statistical temperature that defines the shape of the posterior. This approach has precedent in inverse problems and provides a principled way to avoid underestimating the uncertainty given a single data realization.

With (16), one can then perform a statistical sampling of $E\left(\Theta ,D\right)$ in the *N*-dimensional parameter space. To be self-consistent with the Boltzmann form of the likelihood function, $E\left(\Theta ,D\right)$ was sampled with the Metropolis criteria [34] at a constant temperature 𝒯. Specifically, we used the simulated annealing algorithm [35,36] of Goffe et al. [37] for sampling at a constant 𝒯. Once $𝒫\left(\Theta |D\right)$ is computed, the marginal probability densities $p\left(\Theta_{k}\right)$ can then be obtained by N−1 integrations:

(17) $$p\left(\Theta_{k}\right)=\int d\Theta_{1}d\Theta_{2}\cdots d\;\Theta_{k-1}\cdots d\Theta_{k+1}d\Theta_{N}𝒫\left(\Theta_{1}\;\Theta_{2}\cdots \Theta_{k}\cdots \Theta_{N-1}\Theta_{N}|D\right).$$

In practice, one continues to sample the parameter space until the integrals in (17) converge.

Finally, it is of interest to compare the mathematical structure of the ME approach to a standard Bayesian method. The error function chosen for this analysis was

(18) $$E\left(\Theta \right)={\left(D-M\right)}^{T}\;\left(D-M\right)$$

where D and M are the data and modeled vectors with l multipole l=1,2,⋯L components and T is the transpose. Thus, one can write the ME likelihood as

(19) $$L_{ME}=\exp[-\frac{{\left(D-M\right)}^{T}\;\left(D-M\right)}{𝒯}].$$

In contrast, the Bayesian likelihood is

(20) $$L_{B}\;\sim \exp[-\frac{{\left(D-M\right)}^{T}{\left(C\right)}^{-1}\;\left(D-M\right)}{2}]$$

where C is the LxL data noise covariance matrix, which generally is unknown. If the off-diagonal elements of C are neglected, then

(21) C=ν I

where 𝕀 is the identity matrix and ν is the data variance. Thus, the Bayesian likelihood is equivalent to the ME expression (up to a constant) in the case where it is assumed that the covariance matrix is diagonal and that $\nu =\frac{𝒯}{2}$ = $\frac{<E>}{N}$.

## 3. Model, Parameter Space, and Data Selection for Temperature Anisotropy Analysis

### 3.1. Temperature Anisotropy in the CMB

The quantity of interest in this paper is correlations or temperature anisotropies observed in the CMB that are usually expressed as an average of temperature differences from the mean value $T_{0}$:

(22) $$\Delta T\left(\hat{n}\right)=T\left(\hat{n}\right)-T_{0}=\underset{lm}{\sum }a_{lm}Y_{l}^{m}\left(\hat{n}\right)$$

in two look directions, $\hat{n}$ and ${\hat{n}}^{\prime }$:

(23) $$<\Delta T\left(\hat{n}\right)\Delta T\left({\hat{n}}^{\prime }\right)>=\underset{l}{\sum }\frac{2l+1}{4\;\pi }\;C_{l}P_{l}\left(\hat{n}\;.\;{\hat{n}}^{\prime }\right).$$

Thus, the multipole expansion coefficients are

(24) $$C_{l\;}=\frac{1}{4\;\pi }\;\int d^{2}\hat{n}\;d^{2}{\hat{n}}^{\prime }P_{l}\left(\hat{n}\;.\;{\hat{n}}^{\prime }\right)\;<\Delta T\left(\hat{n}\right)\Delta T\left({\hat{n}}^{\prime }\right)>$$

where $P_{l}$ is the Legendre polynomials and $\delta_{ll^{\prime }}\delta_{mm^{\prime }}C_{l}=<a_{lm}a_{l^{\prime }m^{\prime }}^{*}>$.

The theoretical description of the temperature anisotropies contained in the $a_{lm}$ is centered on correlating them with gauge-invariant adiabatic quantum fluctuations during inflation [38,39,40,41,42]. Required is a physical model of the evolution of these small perturbations with an expanding multi-constituent universe. The two important aspects of this theory are (1) the Einstein field equations for the expansion of the universe and (2) the time evolution and wavenumber-dependent quantum fluctuations of the energy densities of the constituents comprising the universe.

#### 3.1.1. Expansion of the Universe

The key relation derived from general relativity that describes the expansion of an approximate homogeneous and isotropic universe is the Freedman equation, and for a flat universe ($\Omega_{K}\;$ = 0), it is

(25) $$\frac{H\left(z\right)}{H_{0}}=\sqrt{\Omega_{M}{\left(1+z\right)}^{3}+\Omega_{R}{\left(1+z\right)}^{4}+\Omega_{DE}e^{3\int_{0}^{z}\;d\;\ln\left(1+z^{\prime }\right)\left(1+\omega \left(z^{\prime }\right)\right)}}\;$$

where z is the redshift $\left(1+z=\frac{a\left(t_{0}\right)}{a\left(t_{1}\right)}\right)$ and H and $H_{0}$ are the expansion rate $\left(\frac{\frac{d}{dt}a}{a}\right)$ and Hubble constant, respectively. We used the common notation $H_{0}=100\;h$ km/sec/Mpc. The expansion parameter is a, which is evaluated at the present time $t_{0}$ and at an earlier time $t_{1}$. The present-day ratios of density relative to the critical density for matter (hadrons, cold dark matter, leptons (not including neutrinos), and Higgs scalar fields), radiation (photons and neutrinos), and dark energy are $\Omega_{M}$, $\Omega_{R}$, and $\Omega_{DE}$, respectively, with $\Omega_{M}+\Omega_{R}+\Omega_{DE}=1\;≂\;\Omega_{M}+\Omega_{DE}$. It was further assumed that the dark energy density $c^{2}\rho_{DE}\left(z\right)$ obeys an equation of state:

(26) $$𝓅\left(z\right)=\omega \left(z\right)c^{2}\rho_{DE}\left(z\right)\;$$

where $𝓅\left(z\right)$ is the pressure. When $\omega \left(z\right)=-1$ for all z, the last term in the square root in (25) becomes the cosmological constant $\Omega_{\Lambda }$, which is thought to be the vacuum energy of an unknown substance. Komatsu et al. [13] reported ω=−0.98 ≈−1. This value for ω, currently assumed in the ΛCDM model, gives $𝓅\left(z\right)<0,$ which is why cosmologists believe that the present-day universe in a dark-energy-dominant universe (~70 %) is accelerating in its expansion. However, evidence has recently been reported by the Dark Energy Spectroscopic Instrument (DESI) that ω may not be constant [43]. Nevertheless, the current analysis assumed $\omega \left(z\right)=-1$ for all z, as does the ΛCDM model.

#### 3.1.2. Evolution of Quantum Perturbations

It is believed that the quantum perturbations responsible for the observed structure of the CMB and BAO started during the inflation era [42]. At the conclusion of inflation, the universe was cold and dark; it started to reheat as the energy of the vacuum was transferred into radiation and matter. But there are no physical models, such as a non-abelian gauge theory, of how this occurred. Following the reheating era, the early universe consisted primarily of four main constituents: (1) a very dense baryonic plasma consisting of electrons, protons, ions, and neutral atoms; (2) photons; (3) neutrinos; and (4) cold dark matter. The equations governing the mixture of photons and baryonic plasma are generally coupled Boltzmann equations driven by the primordial perturbations.

We require a theory that solves such equations from very early times during inflation through inflation, reheating, and the radiation-, recombination-, and matter-dominated eras and to the present-day dark-energy-dominated era. To this end, paraphrasing Weinberg [38], *for a homogeneous universe for which the quantum perturbations of the Einstein field equations always have a solution, in the limit of a large wavelength, the time-dependent scalar fluctuations of the curvature* $R_{q}\left(t\right)$ *are non-zero and constant in all stages of the evolution of the universe. Further, this condition remains valid whatever the constituents of the universe are; there is always a solution to the field equations for which* $R_{q}$ *is conserved outside the horizon, i.e.,* $\frac{q}{a\left(t\right)}\ll H\left(t\right)$, *and these solutions are referred to as adiabatic.*

$R_{q}$ is conserved outside the horizon in inflation driven by a single scalar field. During inflation, the time-dependent scalar fluctuations of the curvature $R_{q}\left(t\right)$ satisfy the Mukhanov–Sasaki equations [39,40,41], which are integrated from an early time $T_{i}$ during inflation to a time t where $\frac{q}{a\left(t\right)}\ll H\left(t\right)$, allowing one to write the main scalar adiabatic mode in the Newtonian gauge as

(27a) $$\Phi_{q}=\psi_{q}=R_{q}\left[-1+\frac{H\left(t\right)}{a\left(t\right)}\;\int_{T_{i}}^{t}dt^{\prime }a\left(t^{\prime }\right)\right]$$

(27b) $$\delta \rho_{q}\left(t\right)=-\frac{R_{q}\;\dot{\bar{\rho }}\left(t\right)}{a\left(t\right)}\int_{T_{i}}^{t}dt^{\prime }a\left(t^{\prime }\right)$$

(27c) $$\delta u_{q}\left(t\right)=-\frac{R_{q}\;}{a\left(t\right)}\int_{T_{i}}^{t}dt^{\prime }a\left(t^{\prime }\right)\;$$

where $\Phi_{q}\;\mathrm{and}\;\psi_{q}$ are scalar perturbations. In inflation scenarios, $R_{q}$ is parameterized outside the horizon as

(28) $${\left|R_{q}^{0}\right|}^{2}=N^{2}\;q^{-3}{\left(\frac{q/a_{0}}{k_{R}}\right)}^{n_{s}-1}$$

where $N^{2}$, $n_{s}$, and $k_{R}$ are known as the amplitude, the spectral tilt, and a *reference wavenumber*, respectively. The value $n_{s}=1$ refers to a scale-invariant spectrum. The conventional value of $k_{R}\;\mathrm{is}\;0.05$ ${\mathrm{Mpc}}^{-1},$ and due to the details of the normalization, its choice is arbitrary.

#### 3.1.3. Hydrodynamic Model

The ΛCDM model, the standard cosmological model, solves the time evolution of wavenumber-dependent perturbations. It has had unprecedented success in describing many aspects of observational cosmology. For example, both the WMAP group and the Planck collaboration report that there was no clear evidence that the ΛCDM model requires any upgrades. However, for this study, we did not use the ΛCDM model; rather, we used an approximate model to predict the extrema multipoles for the scale power spectrum data. The reason for this was that the high-accuracy model that involves solving coupled Boltzmann equations provides little insight into parameter correlations that, in part, drives uncertainty. Instead, we followed Hu and Sugiyama [24] and Weinberg [25], who developed an approximate semi-analytic *hydrodynamic* model (HM) that permitted a transparent analytic solution for $C_{TT,l}^{S}$.

The equations for the perturbations were first developed in the hydrodynamic limit. Then, solutions were found for the long wavelength limit ($\frac{q}{a\left(t\right)}\ll H\left(t\right),$ far outside the horizon) and also for the short wavelength limit ($\frac{q}{a\left(t\right)}\gg H\left(t\right)$, far inside the horizon). Transfer functions interpolate between these two limits to provide full solutions. The main result for the hydrodynamic model is

(29a) $$\delta_{Dq}=\frac{9\;q^{2}\;t^{2}\;R_{q}^{0}\;T\;\left(k\right)}{10\;a^{2}},$$

(29b) $$\Psi_{q}=-\frac{3\;q^{2}\;tR_{q}^{0}\;T\;\left(k\right)}{5\;a^{2}},$$

(29c) $$\delta_{\gamma q}=\delta_{Bq}=\frac{3\;R_{q}^{0}}{5}\left[T\;\left(k\right)\left(1+3R\right)-{\left(1+R\right)}^{-\frac{1}{4}}\;e^{-\int_{0}^{t}\Gamma \;dt}\;S\left(k\right)\cos\left(\int_{0}^{t}\frac{q\;dt}{a\sqrt{3\left(1+r\right)}}+\Delta \left(k\right)\right)\;\right],$$

(29d) $$\delta u_{\gamma q}=\delta u_{Bq}=\frac{3\;R_{q}^{0}}{5}\left[-t\;T\;\left(k\right)+\frac{a}{\sqrt{3}q{\left(1+R\right)}^{3/4}}e^{-\int_{0}^{t}\Gamma \;dt}\;S\left(k\right)\sin\left(\int_{0}^{t}\frac{q\;dt}{a\sqrt{3\left(1+r\right)}}+\Delta \left(k\right)\right)\;\right],\;$$

where the transfer functions 𝓅, S, and Δ represent the *q* dependence for times well before matter–photon equilibrium to times well after. Expressions for 𝓅, S, and Δ in closed form are provided in [25] and are reproduced in Appendix A and in [17]. The main physics takeaway is that the solutions to the perturbations for the cold dark matter, photons, and baryons are valid from wavelengths that are far outside the horizon to very short wavelengths inside the horizon, and as such, the transfer functions provide the evolution of the perturbation from the primordial universe during inflation to the present day.

Additional approximations concerning the time evolution result in a simple expression for the scalar component of the power spectrum:

(30) $$l\left(l+1\right)C_{TT,\;l}^{S}=\frac{8\;\pi^{2}T_{0}N^{2}\;e^{-2\tau_{reion}}}{25}\int_{1}^{\infty }d\xi \;{\left(\frac{\xi \;l}{l_{R}}\right)}^{n_{s}-1}\;\left\{\frac{1}{\xi^{2}\sqrt{\xi^{2}-1}}\;{\left[3\;T\left(\frac{\xi l}{l_{T}}\right)R_{L}\;-{\left(1+R_{L}\right)}^{-\frac{1}{4}}S\left(\frac{\xi l}{l_{T}}\right)e^{-\frac{\xi^{2}l^{2}}{l_{D}^{2}}}\cos\left(\frac{\xi l}{l_{H}}+\Delta \left(\frac{\xi l}{l_{T}}\right)\right)\right]}^{2}\;+\frac{3\;\sqrt{\xi^{2}-1}}{\xi^{4}\;{\left(1+R_{L}\right)}^{3/2}}\;e^{-\frac{{2\;\xi }^{2}l^{2}}{l_{D}^{2}}}S^{2}\left(\frac{\xi l}{l_{T}}\right)\;\sin^{2}\left(\frac{\xi l}{l_{H}}+\Delta \left(\frac{\xi l}{l_{T}}\right)\right)\right\}$$

where $l_{D}=\frac{d_{A}}{d_{D}},\;l_{T}=\frac{d_{A}}{d_{T}},\;l_{H}=\frac{d_{A}}{d_{H}},\;l_{R}=\left(1+Z_{L}\right)k_{R}d_{A}$, and $d_{T}=\frac{\sqrt[{\;}]{{Ω}_{R}}}{\left(1+Z_{L}\right)H_{0}{Ω}_{M}}=\frac{0.0177}{\Omega_{M}h^{2}}$.

The factor $e^{-2\;\tau_{reion}}$ is an effective damping term that accounts for the effect of the scattering of photons by free electrons, especially following reionization. As a result of the approximations used to derive $l\left(l+1\right)C_{TT,\;l}^{S}$, the prefactor in (30) has the form $N^{2}\exp\left(-2\tau_{reion}\right)$, and thus, there exists an unresolvable ambiguity for $N^{2}$ and $\tau_{reion}$. In the current computation, we fixed $\exp\left(-2\tau_{reion}\right)$ = 0.80209 or $\tau_{reion}=0.110$ [44] and then allowed $N^{2}$ to be one of the free parameters. This value for $\exp\left(-2\tau_{reion}\right)$ was consistent with the value of $\tau_{reion}$ ≂ 0.09 using low-l polarization data from the WMAP collaboration [45] in an analysis of the reionization history of intergalactic matter.

If we define $x=\frac{1}{1+z}$, then the angular distance from Earth to the surface of the last scatter is

(31) $$d_{A}=\frac{c}{H_{0}\left(1+z_{L}\right)}\;\int_{1/\left(1+z_{L}\right)}^{1}dx\;\frac{1}{\sqrt{\Omega_{\Lambda }x^{4}+\Omega_{M}x+\Omega_{R}}}$$

where $z_{L}$ is the redshift at last scatter. The acoustic horizon distance at the last scatter is

(32) $$d_{H}\left(t_{L}\right)=\frac{2}{H_{0}\;\sqrt{3\;R_{L}\;\Omega_{M}\;{\left(1+Z_{L}\right)}^{3/2}}}ln\left(\frac{\sqrt{1+R_{L}}+\sqrt{R_{EQ}+R_{L}}}{1+\sqrt{R_{EQ}}}\right)$$

where $R_{L}=\frac{3\;\Omega_{B}}{4\;\Omega_{\gamma }\;\left(1+Z_{L}\right)}$ is ¾ of the ratio of the baryon-to-photon energy density at the last scatter. The parameter $r_{L}=r\left(t_{L}\right)$ is the distance from a receiver to the surface of the last scatter when $t_{L}$ is about 13.8 Gyr years into the past. It was at this time that the temperature of the universe had cooled sufficiently to allow light to escape the *cosmic soup*. The variables are thus $R_{L}=R\left(t_{L}\right)$ and $a_{L}=a\;\left(t_{L}\right)$. Also, $1+z_{L}$ = 1090, and $R_{EQ}=\frac{3}{4}\frac{\Omega_{R}\;\Omega_{B}}{\Omega_{M}\;\Omega_{\gamma }}$ is R evaluated at the time when there were equal amounts of radiation and matter. Equation (30) includes relations that allow for wavelengths to enter the horizon during the period when the universe was composed of roughly equal parts radiation and matter. Finally, $l_{D}=\sqrt{d_{Landau}^{2}+d_{Silk}^{2}}$ is a damping length. One can see that the CMB multipole spectrum depends on $\Omega_{M}h^{2}$, $\Omega_{B}h^{2}$, $\Omega_{R}h^{2}$, $\Omega_{M}$, $\Omega_{\Lambda }$ and $\Omega_{K}h^{2}$.

The advantage of the HM is that, despite the loss of accuracy as compared to the *standard* ΛCDM model, it contains the essential elements that afford the statistical inference of cosmological parameters from the extrema data. The ΛCDM model has one parameter that is a proxy for a physical parameter, and it is not totally clear why the model works as well as it does. The HM, on the other hand, while not as accurate as the ΛCDM model, is in closed analytic form and affords a clear understanding of the parameter sensitivities describing the multipole spectra of the CMB.

### 3.2. Parameter Space

A basic step in any statistical inference problem is to define the parameter space by identifying parameters that can be varied within the upper and lower bounds, the parameters that are considered fixed, and the parameter constraints. The parameter constraints allow one to *implicitly* infer additional parameter values and uncertainties outside the *N*-dimensional parameter space. The analysis in this paper considered the five-dimensional parameter space $\Theta =\left[n_{s},\;N^{2},\Omega_{M}h^{2}\Omega_{\Lambda },\Omega_{B}h^{2}\right]$. For each point in the 5D space values, h and $\Omega_{M}$ were implicitly computed from the constraints $\Omega_{M}\approx 1-\Omega_{\Lambda }$ and $h=\sqrt{\frac{\Omega_{M}\;h^{2}}{1-\Omega_{\Lambda }}}$. In contrast, the ΛCDM model has a 6D parameter space that, instead of $\Omega_{M}h^{2}$, includes $\Omega_{CDM}h^{2}$, the percentage of cold dark matter in today’s universe. Neglecting the mass of neutrinos, $\Omega_{M}\approx \Omega_{CDM}+\Omega_{B}$. Furthermore, in addition to $\Omega_{CDM}h^{2}$, the ΛCDM model makes an explicit search for $\tau_{reion}$, whereas in the HM model, we fixed $\tau_{reion}=0.110$ from previous analyses of WMAP polarization data, as discussed in Section 3.1.3. The reason that ΛCDM solves for both $\tau_{reion}$ and $\Omega_{CDM}h^{2}$ is that it is solving a complex set of coupled Boltzmann equations that arise when describing the time evolution of the quantum fluctuations, which consist of mostly cold dark matter, driving the acoustic oscillations of the baryonic plasma. Finally, the ΛCDM model has another parameter, $\theta_{*}$, that is not in the HM model and may be viewed as a *proxy parameter* for $r_{*}/{Ɗ}_{A}$, where r_∗_ is the sound horizon at the time of recombination and ${Ɗ}_{A}$ is the comoving angular distance.

The lower bounds (LB) and upper bounds (UB) of the parameter space are shown in Table 1. It was assumed that $p\left(\Theta \right)$ was uniform between the LB and the UB. The uniform priors were consistent with Jaynes’ ME philosophy. In the analysis, the UB and LB were made large enough so that the resulting PDF would satisfy p(x) = 0 for x ≥ UB and $p\left(x\right)=0$ for x ≤ LB.

### 3.3. Data Selection

**Figure 2:**
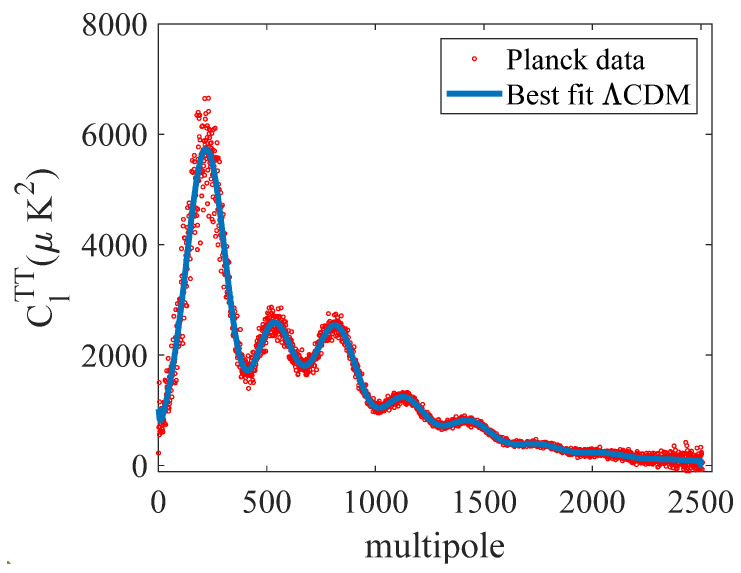
The Planck power spectrum data and the best-fit ΛCDM model have been made available by the European Space Agency: https://irsa.ipac.caltech.edu/data/Planck/release_2/ancillary-data/ (accessed on 6 January 2025).

The data made available are shown in Figure 2. Also made available was the best-fit curve of the data points shown in Figure 1 using the ΛCDM model. The goal in any data-selection process is to balance the benefits of a large band (in the present case, the multipole bandwidth) while minimizing model errors. A subset of the data was chosen where there was a small variance in the selected data points with the best-fit ΛCDM model. Further, the selected data included the reported multipole and amplitude values at the extrema, as reported in [11]. These points were derived by finding the best fit for an assumed Gaussian. Further, to avoid both data and model noise, the points selected for the inversion were in the range of 36<l<1446. The upper value of 1446 was determined from pre-modeling, where, while the HM did a *reasonably good job*, it consistently underpredicted the amplitudes for *l* > 1500.

Once the parameter space and constraints were defined, a statistical inference began by defining $P\left(\Theta \right)$, the model priors. The parameter estimation proceeded as follows: First, the global minimum of the error function $E\left(\hat{\Theta }\right)$ was found in the 5D parameter space. Then, this minimum value of E was used to compute the statistical temperature *𝒯* via the equipartition relation (16). Using this *𝒯*, we applied the Metropolis criteria within a simulated annealing framework at a constant *𝒯 to* sample the parameter space. From the resulting ensemble, we evaluated the posterior probability density $𝒫\left(\Theta |D\right)$ and computed the marginal distributions via integration (17). This process was repeated until the convergence of all the marginal densities was achieved.

## 4. Results

### 4.1. Probability Densities and Parameter Estimates

**Figure 3:**
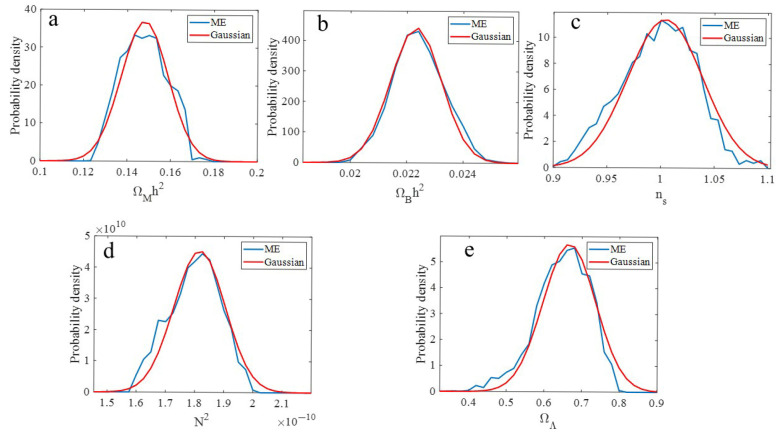
ME and Gaussian best-fit probability densities for (**a**) ${Ω}_{M}h^{2}$, (**b**) ${Ω}_{B}h^{2}$, (**c**) $n_{s}$, (**d**) $N^{2}$, and (**e**) ${Ω}_{\Lambda }$.

Figure 3 shows the probability densities derived from the ME PPD. The distribution $p({Ω}_{B}h^{2})$ exhibited Gaussian-like characteristics, as did $p\left(n_{s}\right)$ and $p\left(\Omega_{M}h^{2}\right)$. In contrast, $p\left(\Omega_{\Lambda }\right)$ had a main peak, but was negatively skewed. The negative skew means that the average value of the inferred value of $\Omega_{\Lambda }$ was less than the value where the peak occurred. To obtain estimates of the parameter mean value and standard deviations, a best-fit Gaussian was plotted with each $p\left(\Theta \right)$. The selection of a Gaussian to extract information from the ME distribution was consistent with the goal of the ME to infer the most conservative estimate of the uncertainty, since a Gaussian is the least informative distribution that maximizes the entropy with constraints for both the mean and the variance.

Table 2 shows the ME Gaussian-inferred parameter values. Included in Table 2 are the optimal $\hat{\Theta }$ parameter values. While *h* and $\Omega_{M}$ are not explicit parameters, one can use the constraints $h=\sqrt{\frac{\Omega_{M}\;h^{2}}{1-\Omega_{\Lambda }}}$ and $\Omega_{M}=1-\Omega_{\Lambda }$ with Monte Carlo sampling to find implicit values to find the mean values and uncertainties. These ME-inferred parameter values are compared to the most recent values reported by the Planck collaboration that fused together multiple data sets and other astrophysical constraints [12]. While the Planck values for $n_{s}$ suggest a small deviation from scale invariance ($n_{s}\;$= 1), the ME values do not support deviations of scale invariance in the primordial density fluctuations. The mean values derived from the ME-inferred parameter $p({Ω}_{B}h^{2})$, which had near Gaussian-like characteristics, agreed well with the Planck values. In contrast, the mean values derived from the ME-inferred parameter $p({Ω}_{M}h^{2})$, which deviated slightly from Gaussian-like characteristics, agreed less well with the Planck values. Furthermore, the mean value derived from the ME-inferred parameter $p({Ω}_{\Lambda })$, which was negatively skewed, was less than those of the Planck values. Because of the constraints, the mean values for *h* and ${Ω}_{M}$ were in reasonable agreement with the Planck values; for example, the ME-derived value for $H_{0}$ was about 67.0 km/sec/Mpc as compared to the Planck value of 67.4 km/sec/Mpc.

The full ME inference process, including the sampling of the 5D parameter space and posterior marginalization, was executed on a single-core CPU system. The total runtime to achieve the convergence of the marginal distributions was about 100 h. Parallelization or GPU-based approaches could significantly reduce this cost.

### 4.2. Modeled and Measured Power Spectra

**Figure 4:**
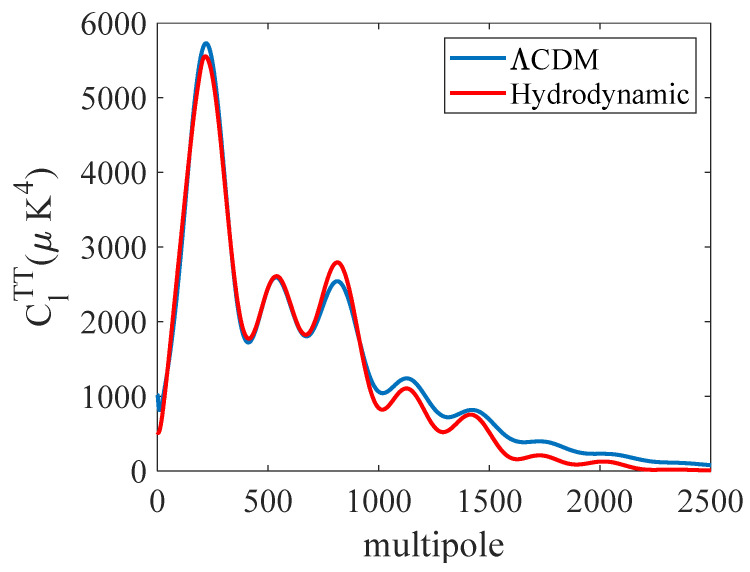
Comparison of the HM $C_{l}^{TT}$ with average parameter values to the best fit made by the *Λ*CDM model. The best-fit ΛCDM power spectrum was obtained online: https://irsa.ipac.caltech.edu/data/Planck/release_2/ancillary-data/ (accessed on 6 January 2025).

Figure 4 compares the $C_{l}^{TT}$ for the ΛCDM and the HM for the average ME Gaussian parameter values in Table 2. The sampling of the error function found parameter values that lowered the error by optimizing the fit to the first and second high-amplitude peaks at the expense of underfitting the power spectra for higher values of *l*. The hydrodynamical model predicted the first two peaks and troughs at about the correct amplitudes. However, the amplitude of the third peak predicted by the HM exceeded that of the ΛCDM prediction and was shifted to slightly higher multipole values.

The main difference between the ΛCDM and the HM were the differences in the amplitude, especially for the higher multipoles, where the HM model tended to underpredict their value. However, if one examines the multipole values in Table 3 where the extrema occurred, the differences between the ΛCDM and HM models were smaller. On average, the ΛCDM slightly underpredicted the measured extrema multipole values, while the HM model slightly overpredicted the multipole values, albeit by a factor of about twice as much as the ΛCDM. This suggests that, if we were to have used the ΛCDM model, the residual error, and thus, the temperature, would be reduced by about a factor of four. Therefore, the probability distribution would be narrower, but not necessarily as narrow as those reported by the Planck collaboration.

## 5. Discussion

Why do the HM and Planck inferences yield a reasonable level of agreement on the $H_{0}$? The short answer is that neither computation infers h explicitly; rather, both infer h implicitly via the constraints $h=\sqrt{\frac{\Omega_{M}\;h^{2}}{1-\Omega_{\Lambda }}}$ and $\Omega_{M}=1-\Omega_{\Lambda }$, where the later constraint is a result of the fact that, at the time of decoupling to the present, the universe has approximately zero curvature. This is an example of how ambiguities in the explicit parameters can cancel in the inference of a parameter that is implicitly determined. This observation strengthens/reaffirms the Planck 2018 case that the value for $H_{0}$ is about 67.4 km/sec/Mpc.

**Figure 5:**
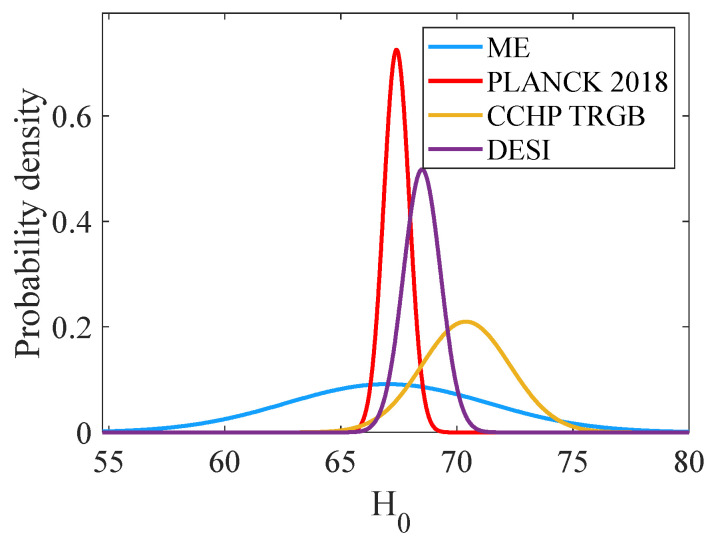
Comparison of the ME Gaussian distributions for $H_{0}$ to probability distributions from the Planck Collaboration [11,12], the Dark Energy Spectroscopic Instrument (DESI) Collaboration [43], and the Carnegie–Chicago Hubble Program (CCHP) using the tip of the red giant branch (TRGB) method measurements [46].

Figure 5 compares the ME Gaussian distributions for *H*_0_ to those obtained with the Kitt Peak DESI and from the CCHP using data collected from the James Webb Space Telescope (JWST). The mean ME value was consistent with the Planck average parameters and within 1σ of the mean values of the DESI and CCHP studies. Due to the conservative nature of the ME approach and the model error, the current analysis did not rule out the possible existence of the so-called Hubble tension, but did not exacerbate it. Since 2024, the CCHP study has decreased its estimate of *H*_0_ from about 72.5 to 70.5 km/sec/Mpc, bringing it closer to the Planck values [46,47]. Thus, one should exercise caution in declaring that there is a Hubble tension, since the analysis of the JWST data is ongoing.

Although the present study did not directly evaluate the $H_{0}$, the ME method offers a robust way to constrain cosmological parameters, including the $H_{0}$ and matter–energy densities, which together define the expansion history of the universe. By propagating uncertainties in a conservative manner, the ME provides a framework that could be extended to yield credible bounds on the cosmic age in future work.

## 6. Conclusions

This paper introduces a fully self-consistent statistical inference approach based on a variant of the Jaynes maximum entropy approach. The method was applied to the scalar component of the cosmic microwave background measurements made by the Planck satellite. A relative entropy function and the equipartition theorem, which provided a statistical temperature, were utilized to derive a posterior probability distribution. The statistical temperature was then used in Metropolis criteria sampling of the PPD. From the PPD, probability densities for the parameter values were then computed. Consistent with the goal of the ME was the use of a Gaussian fit to the probability densities to extract the average and standard deviations for the parameter space. In this sense, in contrast, the ME analysis presented in this study represents a self-consistent approach. A comparison of the Bayesian versus the ME method was presented, with the main difference being how the Bayesian approach estimates the noise covariance matrix, which includes both data and model errors, during the formulation of the likelihood function.

The ME approach adopts the position that the most conservative approach is to make no assumptions about the covariance matrix other than that one can ascertain an average error value. In the current study, one employed the equipartition theorem to relate the average error to a statistical temperature that uniquely specified the likelihood function, and thus, the PPD. This inherently conservative uncertainty reflects the ME approach’s avoidance of assumptions regarding the data covariance matrix, which distinguishes it from conventional Bayesian analyses with narrower error estimates. Once the marginal distributions are inferred, one still has the task of inferring the information content of the probability density functions, and this analysis used a best-fit Gaussian distribution to extract mean values and uncertainties.

This paper contributes to the ongoing Hubble tension controversy by providing an alternative method for estimating cosmological parameters through ME inference rather than the more commonly used Bayesian methods. The ME-inferred value was $H_{0}=67.0$ km/sec/Mpc, which is consistent with the Planck value of 67.4 km/sec/Mpc; however, the ME uncertainty was about 4.3 km/sec/Mpc, compared to the Planck uncertainty value of about 0.05 km/sec/Mpc. These mean values contrast with the mean value of the CCHP study of about 70.4 km/sec/Mpc from redshift data. This discrepancy is at the heart of what has been called the Hubble tension, a major unresolved issue in modern cosmology. Although the ME approach does not definitively resolve the Hubble tension, it provides a valuable perspective. The inferred ME value lies within 1σ of both early-universe estimates (Planck, DESI) and late-universe measurements (e.g., CCHP). Thus, the ME analysis does not appear to support the existence of a tension. It is important to note that the analysis of the JWST data is an ongoing process, where the mean values and uncertainties for $H_{0}$ are still being evaluated. In addition to the Hubble tension, the new DESI measurements place the assumption that ω=−1 in (26) and independent of time into question [43].

The above discussion suggests that a potential future use of the ME approach would be possible extensions of the cosmological model, where one is, for example, testing alternative theories of dark energy and inflation scenarios on uncertain data such as those found in the Sachs–Wolfe region. The idea here is that one would expect model errors, and thus, the ME would conservatively estimate the uncertainty. Given its conservative nature, the ME could serve as a neutral arbiter in comparing models under uncertain data. The ME approach would mitigate accidental bias in the construction of a likelihood for such extensions of the cosmological model.

## Figures and Tables

**Table 1:** Parameter space for hydrodynamic model.

Parameter	Lower Bound	Upper Bound
$n_{s}$	0.90	1.1
$\Omega_{\Lambda }$	0.10	0.90
$\Omega_{M}h^{2}$	0.1	0.2
$\Omega_{B}h^{2}$	0.01	0.03
$N^{2}$	$1.45\times 10^{-10}$	$2.2\times 10^{-10}$

**Table 2:** Comparison of Gaussian fit to ME-inferred parameter probability densities to the Planck 2018 values [12]. The Planck results used a slightly different parameterization for the scalar curvature $R_{q}^{0}$ and it is not straightforward to compare values for $N^{2}$. The Planck values shown here are from the TT, TE, EE+lowE+lensing combination. For a more direct comparison to the scalar-only analysis, the TT+lowE results from Table 2 of Planck 2018 [12] would be more appropriate. The Planck mean value for $\tau_{reion}$ was 0.0544, as compared to the HM value fixed at 0.1100.

Parameter	$\hat{\Theta }$	ME Gaussian	Planck 2018
$n_{s}$	1.0050	1.0040 ± 0.035	0.9649 ±0.0042
$\Omega_{\Lambda }$	0.6649	0.6667 ± 0.070	0.6847 ± 0.0073
$\Omega_{M}h^{2}$	0.1457	0.1480 ± 0.0108	0.1430 ± 0.0011
$\Omega_{B}h^{2}$	0.02243	0.02232 ± 00090	0.02237 ± 0.00015
$10^{10}N^{2}$	1.8208	1.8160 ± 0.0880	—
h	0.6594	0.6700 ± 0.0436	0.6736 ± 0.0054
$\Omega_{M}$	0.3351	0.3333 ± 0.0404	0.3153 ± 0.0073

**Table 3:** Comparison of modeled and measured multipole extrema.

Extrema	ΛCDM	HM	Planck
$P_{1}$	220.8	218.0	220.6
$T_{1}$	410.0	415.5	416.3
$P_{2}$	536.0	541.5	538.1
$T_{2}$	673.0	676.0	675.5
$P_{3}$	814.0	819.0	809.8
$T_{3}$	1017.0	1022.0	1001.1
$P_{4}$	1126.0	1132.0	1147.0
$T_{4}$	1314.0	1300.0	1290.0
$P_{5}$	1421.5	1424.0	1446.8

## Data Availability

The data presented in this study are openly available in https://irsa.ipac.caltech.edu/data/Planck/release_2/ancillary-data/ (assessed on 6 January 2025).

## Appendix A

There are various ways to derive the equipartition theorem. The following was taken from Reif [48]. Consider the energy *E* of a system as a function of *N* canonical coordinates $q_{i}$ and momenta $p_{i}$:

(A1) $$E=E\left(q_{1},\cdots ,q_{N}\;,\;p_{1}\;,\cdots ,p_{N}\right)\;.$$

A guiding principle in both classical and quantum physics is that E separates into

(A2) $$E=\varepsilon \left(p_{i}\right)+\Sigma \left(q_{1}\;,\cdots ,q_{N},p\;,p_{i-1},p_{i+1},\cdots ,p_{N}\right)$$

and that $\varepsilon_{i}$ has the functional form

(A3) $$\varepsilon_{i}\;\left(p_{i}\right)=const\;p_{i}^{2}$$

which immediately allows one to identify *Σ* as a potential. A natural question is as follows: how does one construct $<\varepsilon_{i}>$ in a thermodynamic equilibrium at a temperature 𝒯 if (A1), (A2) are satisfied?

Since the *N* components of the system are distributed by a canonical distribution, (A3) gives a modicum of mathematical steps:

(A4) $$<\varepsilon_{i}>=\frac{\int_{-\infty }^{\infty }dq_{1}\cdots dq_{N}\;dp_{1}\cdots dp_{N}\varepsilon_{i}\;\left(p_{i}\right)\;e^{-\beta \;E\left(q_{1}\;,\cdots ,q_{N}\;,\;p_{1}\;\cdots ,p_{N}\right)}\;}{\int_{-\infty }^{\infty }dq_{1}\cdots dq_{N}\;dp_{1}\cdots dp_{N}e^{-\beta \;E\left(q_{1}\;,\cdots ,q_{N}\;,\;p_{1}\;,\;\cdots ,p_{N}\right)}\;}\;,$$

and from (A2), this becomes

(A5) $$<\varepsilon_{i}>=\frac{\int_{-\infty }^{\infty }dq_{1}\cdots dq_{N}\;dp_{1}\cdots dp_{N}\varepsilon_{i}\;\left(p_{i}\right)\;e^{-\beta \left(\;\varepsilon \left(p_{i}\right)+\Sigma \left(q_{1}\;,\cdots ,q_{N},p\;,p_{i-1},p_{i+1},\;\cdots ,p_{N}\right)\right)}\;}{\int_{-\infty }^{\infty }dq_{1}\cdots dq_{N}\;dp_{1}\cdots dp_{N}e^{-\beta \left(\;\varepsilon \left(p_{i}\right)+\Sigma \left(q_{1}\;,\cdots ,q_{N},p\;,p_{i-1},p_{i+1},\;\cdots ,p_{N}\right)\right)}\;}\;,\;$$

or

(A6) $$<\varepsilon_{i}>=\frac{\int_{-\infty }^{\infty }\;e^{-\beta \;\varepsilon \left(p_{i}\right)}\varepsilon_{i}\;\left(p_{i}\right)dp_{i}\;\int_{-\infty }^{\infty }dq_{1}\cdots dq_{N}\;dp_{1}\cdots dp_{i-1}dp_{i+1}\;\cdots dp_{N}e^{-\beta \;\Sigma }\;}{\int_{-\infty }^{\infty }\;e^{-\beta \;\varepsilon \left(p_{i}\right)}\;dp_{i}\;\int_{-\infty }^{\infty }dq_{1}\cdots dq_{N}\;dp_{1}\cdots dp_{i-1}dp_{i+1}\;dp_{N}e^{-\beta \;\Sigma }\;}$$

The integral terms in the numerator and denominator are common factors:

(A7) $$<\varepsilon_{i}>=\frac{\int_{-\infty }^{\infty }\;e^{-\beta \;\varepsilon \left(p_{i}\right)}\varepsilon \left(p_{i}\right)dp_{i}\;}{\int_{-\infty }^{\infty }\;e^{-\beta \;\varepsilon \left(p_{i}\right)}\;dp_{i}\;}$$

or

(A8) $$<\varepsilon_{i}>=-\frac{\partial }{\partial \beta }\ln(\int_{-\infty }^{\infty }dp_{i}\;e^{-\beta \varepsilon_{i}}),$$

and one easily finds that

(A9) $$<\varepsilon_{i}>=-\frac{\partial }{\partial \beta }\left(-\frac{1}{2}\;\ln\beta \right)=\frac{1}{2\beta }=\frac{1}{2}\;𝒯.$$

Thus, for *N* dimensions, we finally arrive at a relationship between the average energy *E* in an *N*-dimensional space as

(A10) $$<E>=\sum <\varepsilon_{i}>=\frac{N}{2}\;𝒯.$$

For further reading on the equipartition theorem that places it on a firm mathematical foundation, the reader is referred to Tolman [49].

## Appendix B

The transfer functions were constructed by interpolating their values between the limits where $\frac{\rho_{M}}{\rho_{R}}>1$ and $\frac{\rho_{M}}{\rho_{R}}<1$. The arguments of the transfer functions are wavenumber q= $\frac{2\;\pi }{\lambda }$. Weinberg [25] noted that D. Dicus created tables for these transfer functions along with fitting formulas:

$$𝒯\;⋍\;\frac{\ln\left[1+{\left(0.124\;q\right)}^{2}\right]}{{\left(0.124\;q\right)}^{2}}\;{\left[\frac{1+{\left(1.257\;q\right)}^{2}+{\left(0.4452\;q\right)}^{4}+{\left(0.2197\;q\right)}^{6}}{1+{\left(1.606\;q\right)}^{2}+{\left(0.8568\;q\right)}^{4}+{\left(0.3927\;q\right)}^{6}}\right]}^{\frac{1}{2}}$$

$$S\;⋍{\left[\frac{1+{\left(1.209\;q\right)}^{2}+{\left(0.5116\;q\right)}^{4}+\sqrt{5}\;{\left(0.1657\;q\right)}^{6}}{1+{\left(0.9459\;q\right)}^{2}+{\left(0.4249\;q\right)}^{4}+{\left(0.1657\;q\right)}^{6}}\right]}^{2}$$

$$\Delta \;⋍\;{\left[\frac{{\left(1.547\;q\right)}^{2}+{\left(0.5986\;q\right)}^{4}+{\left(0.2578\;q\right)}^{6}}{1+{\left(1.723\;q\right)}^{2}+{\left(0.8707\;q\right)}^{4}+{\left(0.4581\;q\right)}^{6}+{\left(0.2204\;q\right)}^{8}\;}\right]}^{\frac{1}{2}}.$$

In [25], the fitting formula for Δ is missing the square root, as also noted in [17]. In the actual computations, Table 6.1 in [25] was used with a simple interpolation for q<100. For q>100, the fitting formulas were used.
